# Identification of plumericin as a potent new inhibitor of the NF-κB pathway with anti-inflammatory activity *in vitro* and *in vivo*

**DOI:** 10.1111/bph.12558

**Published:** 2014-03-18

**Authors:** N Fakhrudin, B Waltenberger, M Cabaravdic, A G Atanasov, C Malainer, D Schachner, E H Heiss, R Liu, S M Noha, A M Grzywacz, J Mihaly-Bison, E M Awad, D Schuster, J M Breuss, J M Rollinger, V Bochkov, H Stuppner, V M Dirsch

**Affiliations:** 1 Department of Pharmacognosy, University of ViennaVienna, Austria; 2 Department of Pharmaceutical Biology, Faculty of Pharmacy, Universitas Gadjah MadaYogyakarta, Indonesia; 3 Institute of Pharmacy (Pharmacognosy), Center for Molecular Biosciences Innsbruck, University of InnsbruckInnsbruck, Austria; 4 Center for Physiology and Pharmacology, Institute for Vascular Biology and Thrombosis Research, Medical University of ViennaVienna, Austria; 5 Institute of Pharmacy (Pharmaceutical Chemistry), Center for Molecular Biosciences Innsbruck, University of InnsbruckInnsbruck, Austria

**Keywords:** plumericin, NF-κB, inflammation, peritonitis, IκB, IKK-β, natural products, adhesion molecules

## Abstract

**BACKGROUND AND PURPOSE:**

The transcription factor NF-κB orchestrates many pro-inflammatory signals and its inhibition is considered a promising strategy to combat inflammation. Here we report the characterization of the natural product plumericin as a highly potent inhibitor of the NF-κB pathway with a novel chemical scaffold, which was isolated via a bioactivity-guided approach, from extracts of *Himatanthus sucuuba*, an Amazonian plant traditionally used to treat inflammation-related disorders.

**EXPERIMENTAL APPROACH:**

A NF-κB luciferase reporter gene assay was used to identify NF-κB pathway inhibitors from *H. sucuuba* extracts. Monitoring of TNF-α-induced expression of the adhesion molecules VCAM-1, ICAM-1 and E-selectin by flow cytometry was used to confirm NF-κB inhibition in endothelial cells, and thioglycollate-induced peritonitis in mice to confirm effects *in vivo*. Western blotting and transfection experiments were used to investigate the mechanism of action of plumericin.

**KEY RESULTS:**

Plumericin inhibited NF-κB-mediated transactivation of a luciferase reporter gene (IC_50_ 1 μM), abolished TNF-α-induced expression of the adhesion molecules VCAM-1, ICAM-1 and E-selectin in endothelial cells and suppressed thioglycollate-induced peritonitis in mice. Plumericin exerted its NF-κB pathway inhibitory effect by blocking IκB phosphorylation and degradation. Plumericin also inhibited NF-κB activation induced by transfection with the constitutively active catalytic subunit of the IκB kinase (IKK-β), suggesting IKK involvement in the inhibitory action of this natural product.

**CONCLUSION AND IMPLICATIONS:**

Plumericin is a potent inhibitor of NF-κB pathways with a new chemical scaffold. It could be further explored as a novel anti-inflammatory lead compound.

## Introduction

Inflammation represents a response to tissue injury induced by a wide variety of stimuli, including physical damage, infection or alterations caused by malignant cells. As a consequence, many, at first glance, diverse pathological conditions involve inflammatory processes. These include arthritis, atherosclerosis, the metabolic syndrome, sepsis and cancer (Weiss, 2008; Tabas and Glass, 2013). For most of these conditions, no satisfactory treatment is available. Initial stages of inflammation involve cytokine-mediated activation of the vascular endothelium leading to adhesion and transmigration of leukocytes into the site of inflammation. Many of the pro-inflammatory processes elicited at the endothelium and leukocytes are mediated by the transcription factor NF-κB (Perkins, 2007; Rao *et al*., 2007). Major target genes of NF-κB include adhesion molecules (VCAM-1, ICAM-1, E-selectin), cytokines and chemokines (Hayden and Ghosh, 2004). The most prevalent inducers of the NF-κB signalling pathway are cytokines such as TNF-α and IL-1, various mitogens and bacterial components such as LPS.

NF-κB is the name used for a family of homodimers and heterodimers formed of five different transcription factor proteins: p65 (RelA), c-Rel, RelB, p50 and p52. The prototypical complex most often referred to as ‘NF-κB’ is the p65/p50 dimer. The NF-κB dimers are maintained in an inactive state in the cytoplasm bound to the inhibitor of κB (IκB) proteins, of which the prototypical member is IκB-α. Upon a pro-inflammatory signal, such as binding of TNF-α to its membrane receptor, IκB-α becomes phosphorylated at Ser^32^/Ser^36^ by IκB kinase (IKK). The IKK is a multi-subunit kinase complex, most typically composed of IKK-α and IKK-β and two molecules of IKKγ/NEMO (Huynh *et al*., 2000; May *et al*., 2002). The IKK-catalysed phosphorylation triggers degradation of IκB-α leading to the release of NF-κB followed by its translocation to the nucleus where it regulates gene expression. As NF-κB is a key regulator of many pro-inflammatory responses, inhibition of different mediators of the NF-κB signalling pathway, including IKK, has emerged as a promising approach for the development of anti-inflammatory drugs (Karin *et al*., 2004; Gamble *et al*., 2012).

Plant-derived natural products contributed significantly to drug discovery in the past and still provide an effective source for lead structure identification (Newman and Cragg, 2012). Preparations of the stem bark of *Himatanthus sucuuba* have been traditionally used in South America for the treatment of inflammation-associated conditions such as arthritis, cough, ulcer, pain, gastritis and tumors (Amaral *et al*., 2007). Extracts and fractions from *H. sucuuba* latex have been previously shown to suppress carrageenan-induced rat paw oedema (de Miranda *et al*., 2000). The chemical constituents underlying the anti-inflammatory activity and their associated mode of action, however, have not been characterized so far.

In a search for potential NF-κB inhibitors in the stem bark of *H. sucuuba*, this study identified plumericin, a spirolactone iridoid described for the first time from this plant species by Wood *et al*. (2008), as a highly effective novel anti-inflammatory lead compound exhibiting efficacy *in vitro* and *in vivo*.

## Methods

### NF-κB reporter gene assay

HEK293/NF-κB-luc cells (stable HEK293 cell line with a NF-κB-driven luciferase reporter, Panomics, RC0014) were cultured in DMEM (Lonza, Basel, Switzerland) with 2 mM glutamine, 100 μg·mL^−1^ hygromycin B, 100 U·mL^−1^ benzylpenicillin, 100 μg·mL^−1^ streptomycin and 10% FBS. The NF-κB transactivation assay was done as previously described (Rozema *et al*., 2012; Vogl *et al*., 2013). The cells were transfected by the calcium phosphate precipitation method (Graham and van der Eb, 1973) with 4 μg GFP expression plasmid (pEGFP-N1; Clontech, Mountain View, CA, USA). After 6 h, the cells were reseeded in 96-well plates at a density of 4 × 10^4^ cells per well in DMEM containing 0.1% FBS overnight. Cells were pretreated as indicated for 30 min before stimulation with 2 ng·mL^−1^ TNF-α for 4 h. An equal concentration of the solvent vehicle (DMSO, 0.1% or lower) was always included as a control. After cell lysis, the luminescence of the firefly luciferase and the fluorescence of EGFP were quantified on a GeniosPro plate reader (Tecan, Austria). The luciferase-derived signal from the NF-κB reporter was normalized by the EGFP-derived fluorescence to account for differences in the cell number. The known NF-κB inhibitor parthenolide (Bork *et al*., 1997; Lopez-Franco *et al*., 2006) was used as a positive control at a concentration of 5 μM based on published data (Bork *et al*., 1997) and own experiments indicating that parthenolide inhibits NF-κB with an IC_50_ of 1.5 μΜ in our cell model. To analyse the effect of expression of constitutively active IKK-β, 4 μg IKK-β-CA plasmid (IKK-2 S177E/S181E, Addgene-plasmid 11105; Mercurio *et al*., 1997) was cotransfected together with the pEGFP-N1.

### Isolation and identification of compounds from *H. sucuuba*

Powdered bark of *H. sucuuba* (batch number BEL0207) was purchased from Raintree Nutrition, Inc. (Carson City, NV, USA) in July, 2008. Species identity including a certificate of analysis was provided by the supplier. The identity of the plant material was further confirmed by examining the specimen microscopically. A voucher specimen (No. JR-20080730-A1) has been deposited at the Department of Pharmacognosy, University of Innsbruck. Detailed descriptions of the phytochemical work including the isolation and identification of all compounds have been recently provided elsewhere (Waltenberger *et al*., 2011).

### LDH cytotoxicity assay

Quantification of the loss of cell membrane integrity that is associated with cell death can be quantified by the release of the soluble cytosolic protein LDH (Sepp *et al*., 1996; Ehrich and Sharova, 2001). After treatment of cells with the test compounds or solvent vehicle and incubation as indicated in 96-well plates, the supernatant was used to assess the LDH released into the medium. For estimation of the total LDH, identically treated wells were incubated for 45 min in the presence of 1% Triton X-100. The released and total LDH enzyme activity was measured for 30 min at the dark in the presence of 4.5 mg·mL^−1^ lactate, 0.56 mg·mL^−1^ NAD+, 1.69 U·mL^−1^ diaphorase, 0.004% (w/v) BSA, 0.15% (w/v) sucrose and 0.5 mM 2-*p*-iodophenyl-3-nitrophenyl tetrazolium chloride (INT). The enzymic reaction was stopped with 1.78 mg·mL^−1^ oxymate and the absorbance measured at 490 nm. Potential effects on cell viability were estimated as percentage of extracellular LDH enzyme activity. The cytotoxic natural product digitonin (200 μg·mL^−1^) was used as a positive control.

### Analysis of adhesion molecule expression

Immortalized HUVECtert cells (Schiller *et al*., 2009) were maintained in EBM™ growth medium supplemented with 10% FBS, EBM SingleQuots (Lonza, Basel, Switzerland), 100 U·mL^−1^ benzylpenicillin, 100 μg·mL^−1^ streptomycin and 1% amphotericin. The HUVECtert cell line was used instead of primary HUVEC in order to overcome batch-to-batch variability. The cells were seeded at a density of 5 × 10^5^ cells per well overnight in 6-well plates. Then, cells were pretreated with plumericin for 30 min before TNF-α (10 ng·mL^−1^) stimulation for additional 14 h (VCAM-1 and ICAM-1 quantification) or 5 h (E-selectin). Cells were stained with FITC-labelled antibodies [anti-VCAM-1 (BD Biosciences, Schwechat, Austria; 551146), anti-ICAM-1 (eBioscience, Vienna, Austria; BMS108FI), anti-E-selectin (Bio-Rad AbD Serotec, Puchheim, Germany; MCA1969F)] and analysed with a FACSCalibur™ (BD Biosciences) flow cytometer.

### Determination of cAMP levels

HUVECtert were pretreated for 30 min with plumericin (5 μM), solvent vehicle (0.1% DMSO), or forskolin (20 μM) as a positive control. Afterwards, the cells were stimulated with TNF-α (10 ng·mL^−1^) for 10 min and then lysed for 10 min with 0.1 M HCl. The concentrations of cAMP in the cell lysates were determined with *Direct cAMP ELISA kit* (Enzo Life Sciences, Lausen, Switzerland; Catalog #ADI-900-066) using the optional acetylated assay format. The cAMP levels in the test samples were calculated using cAMP standard curves determined in every independent experiment.

### Thioglycollate-induced peritonitis and quantification of neutrophil recruitment

All animal care and experimental procedures were approved by the Animal Experimental Committee of the Medical University of Vienna and by the Austrian Ministry of Science (license no. BMWF-66.009/0117-II/3b/2012). All studies involving animals are reported in accordance with the ARRIVE guidelines for reporting experiments involving animals (Kilkenny *et al*., 2010; McGrath *et al*., 2010). A total of 18 animals were used in the experiments described here.

C57BL/6J male 8–9 weeks old mice were housed in micro-isolation cages and given mouse chow and water *ad libitum* in the mouse care facility at the Institute of Vascular Biology and Thrombosis Research (Vienna, Austria). The mice (5–7 animals per group) were pretreated i.p. with 2 μL plumericin (final concentration 250 μM; dose corresponding to approximately 3 mg·kg^−1^) or DMSO dissolved in 1 mL of saline (0.2% final concentration of DMSO). Thirty minutes later, the animals were injected i.p. with another 1 mL of saline with or without 4% sterile thioglycollate, and containing again 2 μL DMSO or plumericin. Five hours after the second injection, mice were killed by inhaled isofluorane (Baxter, Vienna, Austria), and ice-cold PBS (3 mL) was injected into the abdomen and then collected as i.p. lavage. The volume of the collected lavage was measured and the cell count determined by haemocytometer. The lavage cells were fixed in 4% paraformaldehyde and blocked over night with 5% goat serum in Primary Antibody Diluent (DACO, Vienna, Austria). Alexa Fluor 647 conjugated anti-mouse Ly-6G antibody (eBioscience) and CD11b biotin conjugated antibody (eBioscience) were used for staining. Subsequently, the cells were incubated with Alexa Fluor 488 conjugated streptavidin (Invitrogen, Vienna, Austria) and analysed by flow cytometry (BD FACSCalibur).

### Western blotting

HUVECtert cells (5 × 10^5^ cells per well) were seeded in 6-well plates for 24 h. Cells were pretreated as indicated with plumericin (5 μΜ), SP600125 (50 μΜ), forskolin (20 μΜ), U0126 (10 μΜ) or vehicle and then stimulated with TNF-α (10 ng·mL^−1^) for 10 min. Cell lysates were prepared and used for standard Western blot analysis as described (Baumgartner *et al*., 2010). Anti-IκB-α, anti-phospho-IκB-α, anti-JNK, anti-phospho-JNK (Thr^183^/Tyr^185^), anti-p38, anti-phospho-p38 (Thr^180^/Tyr^182^), anti-ERK1/2 and anti-phospho-ERK1/2 (Thr^202^/Tyr^204^) antibodies were purchased from Cell Signalling Technology (Danvers, MA, USA), the anti-tubulin antibody was obtained from Santa Cruz Biotechnology (Santa Cruz, CA, USA) and the anti-actin antibody from MP Biomedicals (Illkirch, France). All antibodies were used in a dilution of 1:1000.

### Immunoprecipitation and enzyme activity of the endogenous IKK complex

HUVECtert cells (2.2 × 10^6^ cells per well) were seeded in 10 cm dishes for 48 h. The cells were pretreated as indicated with plumericin or solvent vehicle (0.1% DMSO) for 30 min and then stimulated with TNF-α (10 ng·mL^−1^) for 10 min. Cells were washed with PBS and lysed at 4°C with by gentle swirling in a buffer containing 10% glycerol, 1 mM EDTA, 100 μg·mL^−1^ digitonin, 40 mM NaCl, 50 mM KCl, 20 mM Tris pH 7.4, 0.1% Triton X-100, 2 mM DTT, 1 mM Na_3_VO_4_, 0.5 mM NaF and 1 mM PMSF. Afterwards, cell debris was removed by centrifugation for 10 min at 3000× *g* and the supernatants were swirled with 6 μg anti-IKKγ/NEMO antibody (Santa Cruz, Heidelberg, Germany) for 1.5 h at 4°C. 50 μL of 50% protein A agarose beads suspension was then added and the supernatants were rotated for further 45 min. Afterwards, the beads were washed two times with 1 mL of lysis buffer, and three more times with 1 mL from a buffer containing 25 mM HEPES pH 7.4, 2 mM MgCl_2_, 2 mM MnCl_2_ and 63 μM ATP. The enzymic activity of the immunoprecipitated IKK was determined with CycLex IKK α and β Assay/Inhibitor Screening Kit (MBL International, Woburn, MA, USA; Cat# CY-1178) according to the instructions of the manufacturer.

### Kinase assay with recombinant IKK-β

The kinase activity of human recombinant IKK-β was measured by the elisa-based (K-LISA™) IKK-β activity assay (Millipore/Calbiochem, Vienna, Austria) as previously described (Noha *et al*., 2011). Briefly, the test compound or solvent vehicle (0.1% DMSO) were incubated for 30 min at 30°C with the recombinant IKK-β and a substrate peptide (GST-IκB-α 50-amino acid peptide including Ser^36^ and Ser^32^ IKK-β phosphorylation sites) in a glutathione-coated 96-well plate, allowing substrate phosphorylation and capture in a single step. The phosphorylated GST-IκB-α substrate was finally detected using anti-phospho(Ser^32^/Ser^36^)-IκB-α antibody (Cell Signalling Technology), followed by the HRP-conjugated secondary antibody. The colour development of the HRP substrate was measured at 450 nm on a Tecan GeniosPro plate reader (Tecan, Grödig, Austria).

### Novelty evaluation

The novelty evaluation of the findings was performed with PubMed and SciFinder searches with ‘plumericin’ as keyword, resulting in 19 and 85 literature references respectively (on 18 July 2013). None of the retrieved references was related to NF-κB modulation. To further evaluate the novelty of the chemical scaffold of plumericin as an inhibitor of NF-κB, chemical similarity search was performed with SciFinder, using the structure of plumericin with threshold set at above 70% Tanimoto similarity scores. As outcome, 169 similar chemical structures, associated with 226 literature references, were retrieved. None of the obtained literature references was related to NF-κB.

### Data analyses

Data in the Figures represent means ± SEM. Statistical analyses were performed using GraphPad Prism® 4.03. Non-linear regression (sigmoidal dose response) was used to calculate the IC_50_ values. Statistical differences among the treatment groups were compared using one-way anova. *P*-values < 0.05 were considered significant.

### Materials

The following chemicals were supplied by Sigma-Aldrich (Vienna, Austria): 2-*p*-iodophenyl-3-nitrophenyl tetrazolium chloride (INT), digitonin and TNF-α. SP600125 was supplied by Tocris (Bristol, UK) and forskolin by Enzo Life Sciences.

## Results

### Plumericin isolated from *H. sucuuba* is a potent inhibitor of the NF-κB pathway

**Figure 1:**
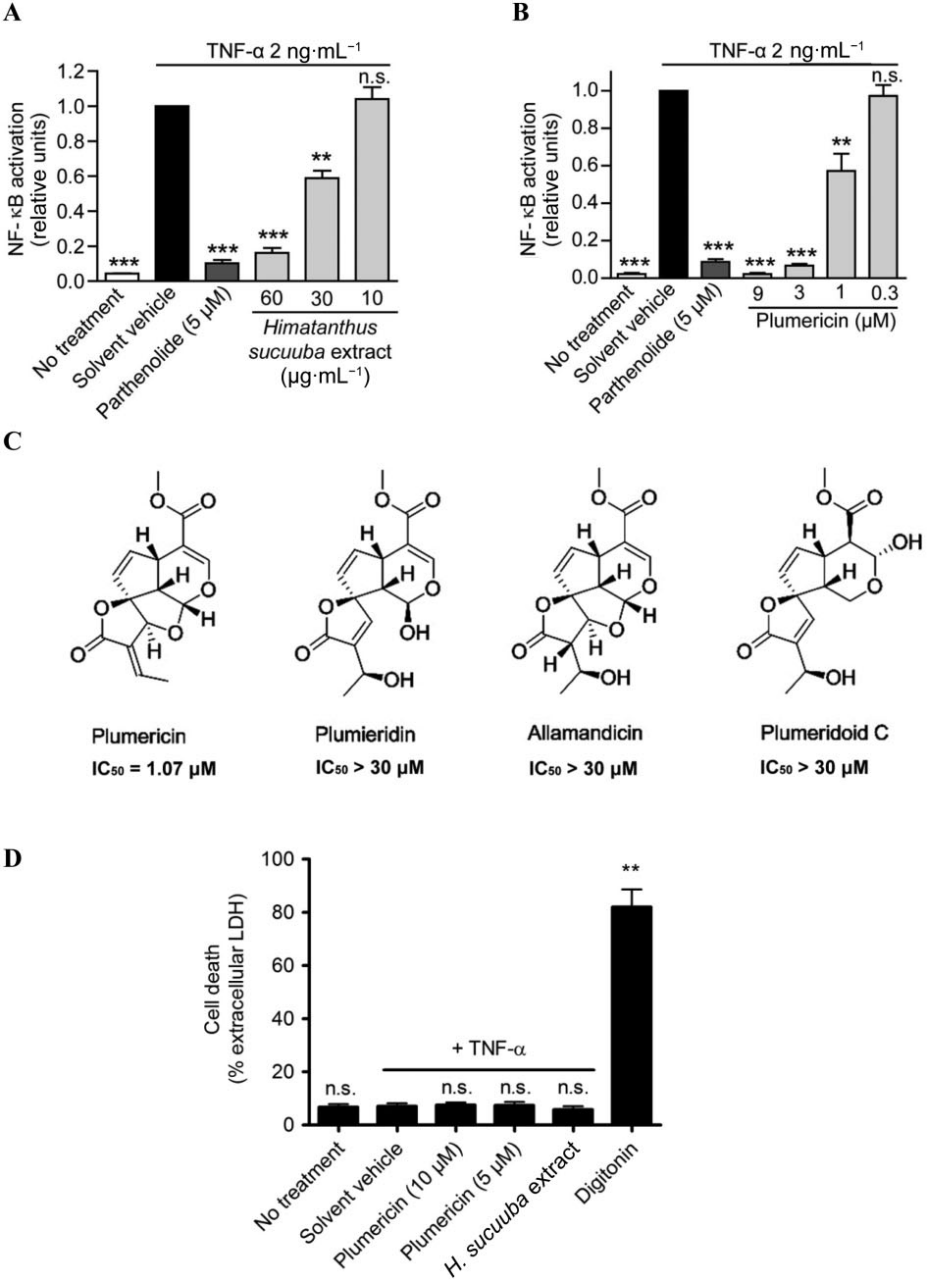
An ethyl acetate extract of *H. sucuuba* and the compound plumericin inhibit TNF-α-induced NF-κB activation. HEK293/NF-κB-luc cells were pretreated for 30 min with the indicated concentrations of ethyl acetate extracts of *H. sucuuba* bark (A), plumericin (B), solvent vehicle (DMSO 0.1%) or 5 μM parthenolide as positive control, prior to stimulation with 2 ng·mL^−1^ TNF-α for 4 h. (C) Chemical structures of the potent NF-κB inhibitor plumericin and the other isolated, structurally similar, compounds that were inactive. (D) Cytotoxicity determination in the presence of plumericin or *H. sucuuba* extract. HEK293/NF-κB-luc cells were treated as indicated in A and B with solvent vehicle, plumericin (5 or 10 μM), *H. sucuuba* extract (60 μg·mL^−1^), or digitonin (200 μg·mL^−1^) as positive control. LDH enzyme activity was determined 4 h after the TNF-α stimulation. Data shown are means ± SEM (*n* = 3. ***P <0.001,**P < 0.01, n.s. not significant, anova with Dunnett's *post hoc* test).

In order to detect inhibitory effects on the NF-κB pathway, we first tested an ethyl acetate extract of the stem bark of *H. sucuuba* and observed a dose-dependent suppression of TNF-α-induced NF-κB activation in a NF-κB-driven luciferase reporter model (Figure 1A). The NF-κB-inhibitory potential of the extract was confirmed in immortalized HUVECtert (Schiller *et al*., 2009) by its ability to suppress the TNF-α-or LPS-induced expression of the NF-κB target genes E-selectin and IL-8 (over 80% suppression at 50 μg·mL^−1^, data not shown). Bioactivity-guided fractionation of the extract resulted in the isolation and identification of the highly active novel NF-κB pathway inhibitor plumericin (Figure 1B,C) together with other compounds with less or no activity, plumieridin, allamandicin, plumeridoid C, biochanin A, dihydrobiochanin A, dalbergioidin, ferreirin, dihydrocajanin, naringenin and pinoresinol. The isolation and identification of these compounds has been fully described by Waltenberger *et al*., (2011), and direct comparison of their NF-κB inhibitory potential is shown in Supporting Information Fig. S1. The spirolactone iridoid plumericin inhibited TNF-α-induced NF-κB activation with an IC_50_ of 1.07 μΜ. The *H. sucuuba* extract (60 μg·mL^−1^), plumericin (5 μΜ), and the positive control parthenolide (5 μΜ) had no significant effect on the basal NF-κB activity in the absence of TNF-α stimulation (Supporting Information Fig. S2). No cytotoxicity was observed by quantification of cell membrane integrity measuring LDH release after exposure of the cells for 4 h to plumericin, up to 10 μΜ or *H. sucuuba* extract, up to 60 μg·mL^−1^ (Figure 1D). In the concentration range up to 10 μΜ, plumericin did not exhibit cytotoxicity also upon prolonged exposure time (24 h), although under this experimental condition, higher concentrations of plumericin (30 μΜ) induced significant loss of cell viability (Supporting Information Fig. S3).

Plumericin has a chemical scaffold clearly different from other previously described inhibitors of the NF-κB pathway (see ‘Novelty evaluation’ section in the ‘Methods’ chapter). Interestingly, three other structurally related iridoid lactones isolated from *H. sucuuba*: plumieridin, allamandicin and plumeridoid C (Figure 1C), did not inhibit the TNF-α-induced NF-κB-luciferase reporter transactivation in concentrations up to 30 μΜ. Structure-activity comparison of the four closely related molecules disclosed the importance of the exocyclic α-methylene-γ-lactone group present only in plumericin and not in the other molecules (Figure 1C).

### Plumericin abolishes the induction of adhesion molecules in endothelial cells and leukocyte recruitment to inflamed peritoneum in mice

**Figure 2:**
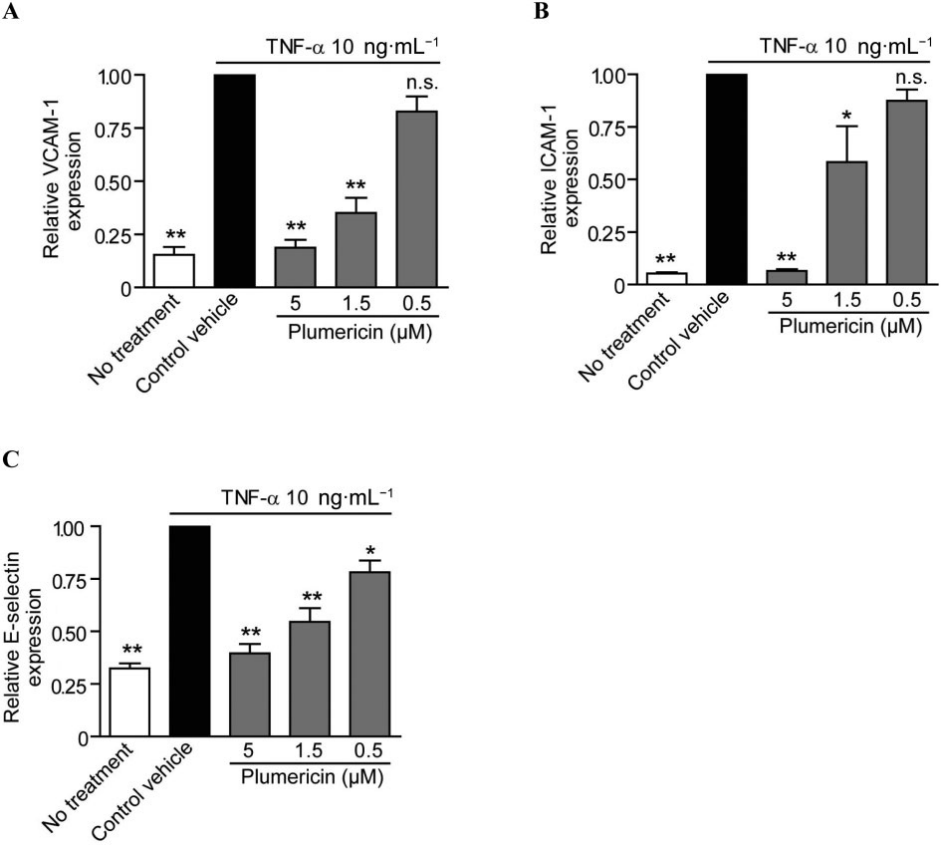
Plumericin inhibits TNF-α-induced cell surface expression of the endothelial adhesion molecules VCAM-1 (A), ICAM-1 (B) and E-selectin (C). HUVECtert cells were pretreated with the indicated concentrations of plumericin or solvent vehicle as control (DMSO 0.1%) for 30 min prior to stimulation with 10 ng·mL^−1^ TNF-α for 14 h (VCAM-1, ICAM-1) or 5 h (E-selectin). The protein expression levels were analysed by flow cytometry. Data shown are means ± SEM (*n* = 3; *P < 0.05, **P < 0.01, n.s. not significant, anova with Dunnett's *post hoc* test).

**Figure 3:**
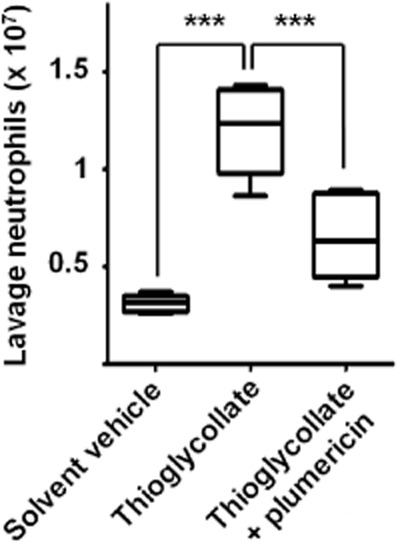
Plumericin inhibits neutrophil recruitment in a thioglycollate-induced peritonitis model. C57BL/6 male mice (5–7 animals per group) were pretreated i.p. with 2 μL plumericin (final concentration 250 μM) or DMSO dissolved in 1 mL of saline. Thirty minutes later, the animals were injected i.p. with 1 mL of solvent vehicle or 4% sterile thioglycollate containing 2 μL DMSO or plumericin. Five hours after the second injection, mice were killed. Peritoneal lavage fluid was collected, and the neutrophil number was quantified by flow cytometry. The graph shows box and whisker plots representing median, upper and lower quartile, and lowest and highest detected values (***P < 0.001, anova with Dunnett's *post hoc* test).

The inhibitory effects of plumericin were confirmed in a cell model more physiologically relevant for the pro-inflammatory activity of NF-κB, by studying the effects of this compound on the expression of the adhesion molecules VCAM-1, ICAM-1 and E-selectin induced by TNF-α in endothelial cells. These three adhesion molecules are well known NF-κB target genes that are upregulated in the endothelium during inflammation (Hayden and Ghosh, 2004). In line with the results from the NF-κB-driven luciferase reporter model (Figure 1B), plumericin suppressed potently and dose-dependently the TNF-α-induced expression of VCAM-1, ICAM-1 and E-selectin in HUVECtert cells (Figure 2A–C). Furthermore, as in the NF-κB-driven luciferase reporter model (Figure 1B and Supporting Information Fig. S1), the structurally related plumieridin, allamandicin and plumeridoid C did not inhibit the TNF-α-induced expression of the NF-κB target genes VCAM-1, ICAM-1 and E-selectin (Supporting Information Fig. S4), and none of the investigated compounds interfered with the basal levels of these adhesion molecules in the absence of TNF-α stimulation (Supporting Information Fig. S5). As adhesion molecule expression on the endothelium is a prerequisite for the recruitment of leukocytes from the blood to the site of inflammation (Rao *et al*., 2007), we further tested whether plumericin would reduce polymorphonuclear leukocyte recruitment *in vivo* using a thioglycollate-induced peritonitis mouse model. As shown in Figure 3, plumericin did inhibit recruitment of neutrophils to the inflamed peritoneum of mice with thioglycollate-induced peritonitis.

### Plumericin inhibits the NF-κB pathway through selective inhibition of the IKK-mediated phosphorylation and degradation of IκB

**Figure 4:**
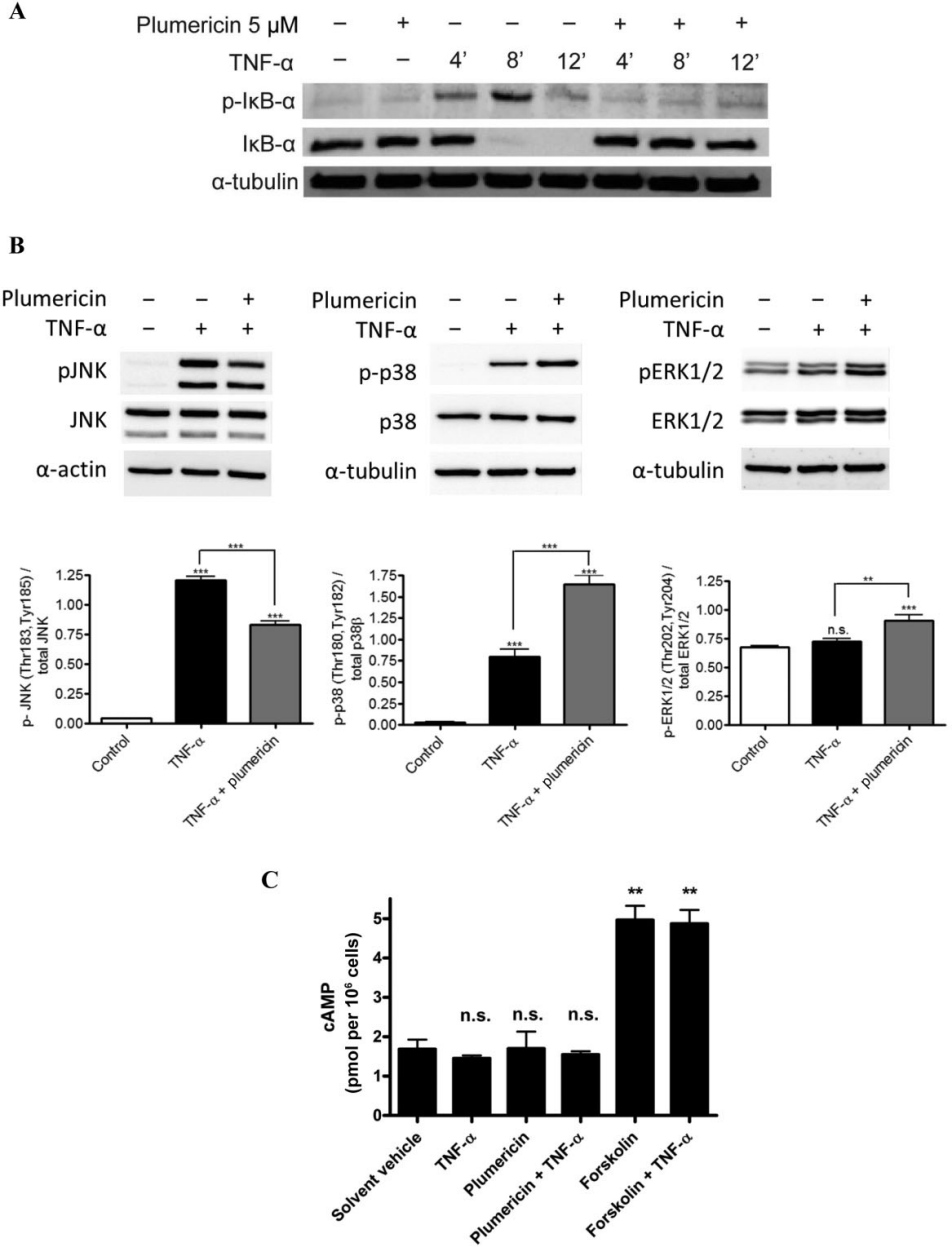
Plumericin inhibits the NF-κB pathway through inhibition of the IKK-mediated phosphorylation and degradation of IκB. (A) HUVECtert cells were pre-incubated with plumericin (5 μΜ) or solvent vehicle (DMSO, 0.1%) for 30 min prior to stimulation with TNF-α for 0, 4, 8 or 12 min. Western blot analysis was performed for α-tubulin, IκB-α and phospho(Ser^32^/Ser^36^)-IκB-α (p-IκB-α). Representative blots out of three independent experiments are shown. (B) HUVECtert cells were pre-incubated with plumericin (5 μΜ) or solvent vehicle (DMSO, 0.1%) for 30 min prior to stimulation with TNF-α (10 ng·mL^−1^) for 10 min. Western blot analysis was performed for the total and phosphorylated JNK, p38 and ERK1/2 as described in the Methods section. Tubulin or actin was used as a loading control. Results from each set of blots are summarised in the bar graphs; data shown are means ± SD. *n* = 3, ***P < 0.001, **P < 0.01, n.s. not significant, anova with Bonferroni's *post hoc* test. (C) HUVECtert cells were pre-incubated with plumericin (5 μΜ), forskolin (20 μΜ) as positive control or solvent vehicle (DMSO, 0.1%) for 30 min prior to stimulation with TNF-α (10 ng·mL^−1^) for 10 min. Cells were lysed and cAMP levels determined by elisa. The data presented are from four independent experiments and are means ± SEM. **P < 0.01, n.s. not significant, anova with Dunnett's *post hoc* test.

A key step in the NF-κB signalling pathway is the degradation of IκB, which leads to release of bound NF-κB for subsequent nuclear translocation. Therefore, we first studied the influence of plumericin on TNF-α-induced degradation of IκB-α in HUVECtert cells. IκB-α was rapidly degraded upon stimulation with TNF-α, and plumericin (5 μM) completely inhibited this degradation process (Figure 4A). The degradation of IκB-α upon TNF-α stimulation is induced by IKK-dependent Ser^32^/Ser^36^ phosphorylation of IκB-α. Accordingly, a strong increase in the phosphorylation of these residues was observed shortly before the complete degradation of IκB-α after TNF-α treatment. In the presence of plumericin, IκB-α phosphorylation was abolished, indicating reduced activity of IKK (Figure 4A). To examine whether plumericin also affects the activity of other kinases activated by TNF-α in HUVECtert cells, we further studied the phosphorylation status of JNK, p38 and ERK1/2 (Figure 4B). While TNF-α induced the phosphorylation of JNK and p38, the phosphorylation of ERK1/2 did not increase above the basal level, and plumericin (5 μM) had no potent inhibitory effect on the TNF-α-induced phosphorylation pattern of any of the three kinases studied (Figure 4B). The TNF-α-induced JNK phosphorylation was inhibited by 25%, whereas the phosphorylation of p38 and ERK1/2 was increased in the presence of plumericin. As reference inhibitors for the investigated phosphorylation sites (Supporting Information Fig. S6), we used 50 μΜ SP600125 to inhibit the phosphorylation of JNK at Thr^183^/Tyr^185^ (Bennett *et al*., 2001), 20 μΜ forskolin to inhibit the phosphorylation of p38 at Thr^180^/Tyr^182^ (Rahman *et al*., 2004) and 10 μΜ U0126 to inhibit the phosphorylation of ERK1/2 at Thr^202^/Tyr^204^ (Wang *et al*., 2006). To investigate whether plumericin could interfere with cAMP levels, which are a relevant determinant of NF-κB activation (Jin *et al*., 2005; Gerlo *et al*., 2011; Yougbare *et al*., 2013), we quantified cAMP in HUVECtert cells exposed to plumericin (Figure 4C). Under the same treatment conditions, in which plumericin (5 μM) abolished TNF-α-induced IκB-α phosphorylation and degradation (Figure 4A), no changes in intracellular cAMP levels were observed in the presence or absence of plumericin, with or without TNF-α treatment. Thus, we can exclude an interference with cAMP metabolism as a likely explanation of the effects observed.

**Figure 5:**
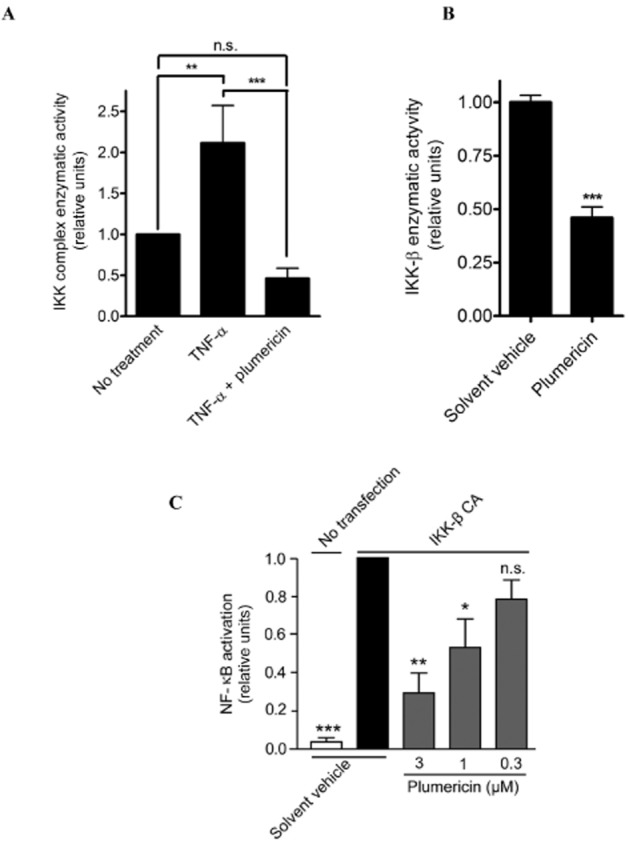
Plumericin-mediated inhibition of the NF-κB pathway involves IKK. (A) HUVECtert were pretreated with plumericin (10 μM) or solvent vehicle (0.1% DMSO) for 30 min and then stimulated with TNF-α (10 ng·mL^−1^) or with vehicle (cell culture medium) for 10 min. IKK complex was immunoprecipitated with anti-IKKγ/NEMO antibody and protein A agarose beads, and its enzymic activity was determined. Data shown are means ± SEM. *n* = 3, ***P < 0.001, **P < 0.01, n.s. not significant, anova/Bonferroni. (B) The enzyme activity of human recombinant IKK-β was determined for 30 min at 30°C in the presence of solvent vehicle (0.1% DMSO) or plumericin (10 μM). The phosphorylation of the Ser^32^/Ser^36^-IκB-α substrate peptide was detected by elisa. Data shown are means ± SEM. *n* = 3; ***P < 0.001, unpaired two-tailed *t*-test. (C) HEK293/NF-κB-luc cells were transfected with constitutively active IKK-β (IKK-β-CA) by the calcium phosphate precipitation method, incubated overnight and treated with the indicated concentrations of plumericin for 4 h. Data shown are means± SEM. *n* = 3; *P < 0.05, **P < 0.01, ***P < 0.001, n.s. not significant, anova with Dunnett's *post hoc* test.

To confirm a role for IKK in the plumericin-mediated NF-κB pathway inhibition, we determined the effect of plumericin on the enzymic activity of the endogenous IKK complex immunoprecipitated from HUVECtert cells. Treatment with plumericin (10 μM) abolished the TNF-α-induced IKK activation, restoring it to basal levels (Figure 5A). Plumericin also suppressed the enzymic activity of human recombinant IKK-β (Figure 5B). We further performed transfection experiments with a constitutively active form of the IKK catalytic subunit IKK-β (IKK-β-CA; Mercurio *et al*., 1997), which is known to cause IKK activation independently of upstream signalling events. Transfection of IKK-β-CA into HEK293/NF-κB-luc cells indeed resulted in a strong stimulus-independent activation of NF-κB (Figure 5C). The inhibitory action of plumericin in this setting, with close to 50% signal reduction at 1 μΜ (Figure 5C) was very similar to the inhibition observed following TNF-α stimulation (Figure 1B, IC_50_ of 1.07 μΜ) pointing to IKK as a direct target of plumericin.

## Discussion and conclusions

In a search for novel inhibitors of the NF-κB pathway from natural sources, we investigated an extract from the stem bark of *H. sucuuba*, a medicinal plant traditionally used in South America to treat different inflammation-related diseases. Bioactivity-guided fractionation led to the identification of the spirolactone iridoid plumericin as a potent inhibitor of the NF-κB pathway, with a new chemical scaffold.

Plumericin effectively abolished the NF-κB activation in a luciferase reporter cell model (Figure 1B), inhibited TNF-α-induced expression of the pro-inflammatory adhesion molecules VCAM-1, ICAM-1 and E-selectin in endothelial cells (Figure 2A–C), and suppressed neutrophil recruitment to the peritoneum in response to thioglycollate in mice (Figure 3). Analysis of the molecular mechanism of action of plumericin revealed that this compound abolished NF-κB pathway signalling through inhibition of the IKK-mediated phosphorylation and subsequent degradation of IκB (Figure 4A), without a strong inhibitory impact on the TNF-α-induced phosphorylation pattern of the kinases JNK, p38 and ERK1/2 (Figure 4B). Interestingly, the structure-activity comparison of plumericin with the inactive derivatives plumieridin, allamandicin and plumeridoid C (Figure 1C) revealed the importance of the α-methylene-γ-lactone group present only in plumericin, for its inhibitory action on the NF-κB pathway inhibition. In line with our findings, the α-methylene-γ-lactone functional moiety is also described to be important for the action of several previously described IKK inhibitors (Kwok *et al*., 2001; Huang *et al*., 2004). Plumericin suppressed the activity of the endogenous IKK complex immunoprecipitated from HUVECtert cells (Figure 5A) and inhibited the activity of human recombinant IKK-β (Figure 5B). Furthermore, plumericin abolished the NF-κB activation induced by overexpression of the constitutively active catalytic IKK subunit, IKK-β (Figure 5C), with a potency very similar to that observed upon TNF-α stimulation (Figure 1B), confirming IKK as a direct target within the NF-κB pathway.

The effect of plumericin applied at 3 mg·kg^−1^ in the thioglycollate-induced peritonitis model (Figure 3) appears slightly stronger than the described effect of the selective IKK inhibitor IKK 16 which inhibited neutrophil extravasation in this model to about 50% at a dose of 10 mg·kg^−1^ (Waelchli *et al*., 2006). The effect of plumericin was also comparable to that of the known NF-κB inhibitor, parthenolide, blocking neutrophil recruitment upon intra-tracheal LPS challenge at similar concentrations (Saadane *et al*., 2012). However, another well-established NF-κB inhibitor, BAY11-7085, was effective only at a 10-fold higher concentration in two *in vivo* models of inflammation, the rat carrageenan paw model and rat adjuvant arthritis (Pierce *et al*., 1997). Thus, plumericin inhibited inflammation *in vivo* at concentrations comparable to, or lower than, those of existing NF-κB inhibitors.

Whereas diverse plant-derived compounds (Nam, 2006) such as polyphenols, lignans and terpenoids have been demonstrated to suppress signalling in the NF-κB pathway, in higher concentrations and usually *in vitro*, the exceptional potency and *in vivo* efficacy of plumericin rank this compound among the most interesting natural products so far described as NF-κB pathway blockers.

The identification of plumericin as a potent new inhibitor of the NF-κB pathway provides a solid scientific rationale for the traditional use of extracts of *H. sucuuba* as herbal medicines for the treatment of inflammatory diseases. Its new chemical scaffold warrants further exploitation of plumericin as a possible lead structure for the development of new anti-inflammatory drugs.

## Abbreviations

IKK: IκB kinase
INT: 2-*p*-iodophenyl-3-nitrophenyl tetrazolium chloride

## Supporting information

Figure S1 Among all isolated compounds only plumericin is a potent inhibitor of TNF-α-induced NF-κB activation. HEK293/NF-κB-luc cells were pretreated for 30 min with the indicated compounds (10 μMeach) or solvent vehicle (DMSO 0.1%), and stimulated with 2 ng·mL^-1^ TNF-α for 4 h. The data represent mean ± SEM (*n* = 3; *P < 0.05, **P < 0.01, n.s. not significant, ANOVA/Dunnett).

Figure S2 Comparison of the effects of plumericin, parthenolide, and the H. *sucuuba* extract on NF-κB activity in the presence and absence of TNF-α stimulation. HEK293/NF-κBluc cells were pretreated for 30 min with H. *sucuuba* extract (60 μg·mL^-1^), plumericin (5 μμ), parthenolide (5 μμ), or solvent vehicle (DMSO 0.1%), and stimulated with 2 ng·mL^-1^ TNF-α or treated with the same volume of vehicle (cell culture medium) for 4 h. The data represent mean ± SEM (*n* = 3; ***P < 0.001, n.s. not significant, ANOVA/Bonferroni).

Figure S3 Cytotoxicity determination upon prolonged exposure and in the presence of higher concentrations of plumericin. HEK293/NF-κB-luc cells were treated as indicated in Figure 1A and Figure 1B with solvent vehicle (0.1% DMSO), different concentrations of plumericin, or digitonin (200 μg·mL^-1^) as positive control. LDH enzyme activity was determined 24 h after the TNF-α stimulation. All data represent mean ± SEM (*n* = 3; **P < 0.01, n.s. not significant, ANOVA/Dunnett).

Figure S4 Plumericin but not the other structurally related compounds isolated from H. *sucuuba* inhibits TNF-α-induced cell surface expression of the endothelial adhesion molecules VCAM-1, ICAM-1, and E-selectin. HUVECtert cells were pretreated with 10 μμ plumericin, parthenolide, plumieridin, allamandicin, plumeridoid C or solvent vehicle (DMSO 0.1%) as control for 30 min prior to stimulation with 10 ng·mL^-1^ TNF-α for 14 h (VCAM-1, ICAM-1) or 5 h (E-selectin). The protein expression levels were analysed by flow cytometry. Data shown are mean ± SEM (n = 3 for VCAM-1 and ICAM-1, *n* = 4 for E-selectin; *P < 0.05, **P < 0.01, n.s. not significant, ANOVA/Dunnett).

Figure S5 Plumericin and the other investigated compounds do not affect the basal expression of the endothelial adhesion molecules VCAM-1, ICAM-1, and E-selectin in the absence of TNF-α stimulation. HUVECtert cells were treated with 10 μμ plumericin, parthenolide, plumieridin, allamandicin, plumeridoid C or 10 ng·mL^-1^ TNF-α for 14 h (VCAM-1, ICAM-1) or 5 h (E-selectin). The protein expression levels were analysed by flow cytometry. Data shown are mean ± SEM (*n* = 3; **P < 0.01, n.s. not significant, ANOVA/Dunnett).

Figure S6 Effect of reference inhibitors on the investigated phosphorylation sites of JNK, p38, and ERK1/2. HUVECtert cells were pre-incubated for 30 min with SP600125 (50 μμ), forskolin (20 μμ), U0126 (10 μμ) or solvent vehicle (DMSO, 0.1% or 0.2%, respectively), prior to stimulation with TNF-α (10 ng·mL^-1^) for 10 min.Western blot analysis was performed for the total and phosphorylated JNK, p38, and ERK1/2 as described in the Methods section. Tubulin or actin was used as a loading control. Representative blots out of three independent experiments and their quantification as bar graphs (*n* = 3, mean ± SD, *P < 0.05, ***P < 0.01, n.s. not significant, ANOVA/Bonferroni) are shown.
