# Supplementary figures and images for: Identification of plumericin as a potent new inhibitor of the NF-κB pathway with anti-inflammatory activity in vitro and in vivo
Source: Br J Pharmacol. 2014 Mar 18;171(7):1676–86. doi: 10.1111/bph.12558 (PMC3966748; doi:10.1111/bph.12558)

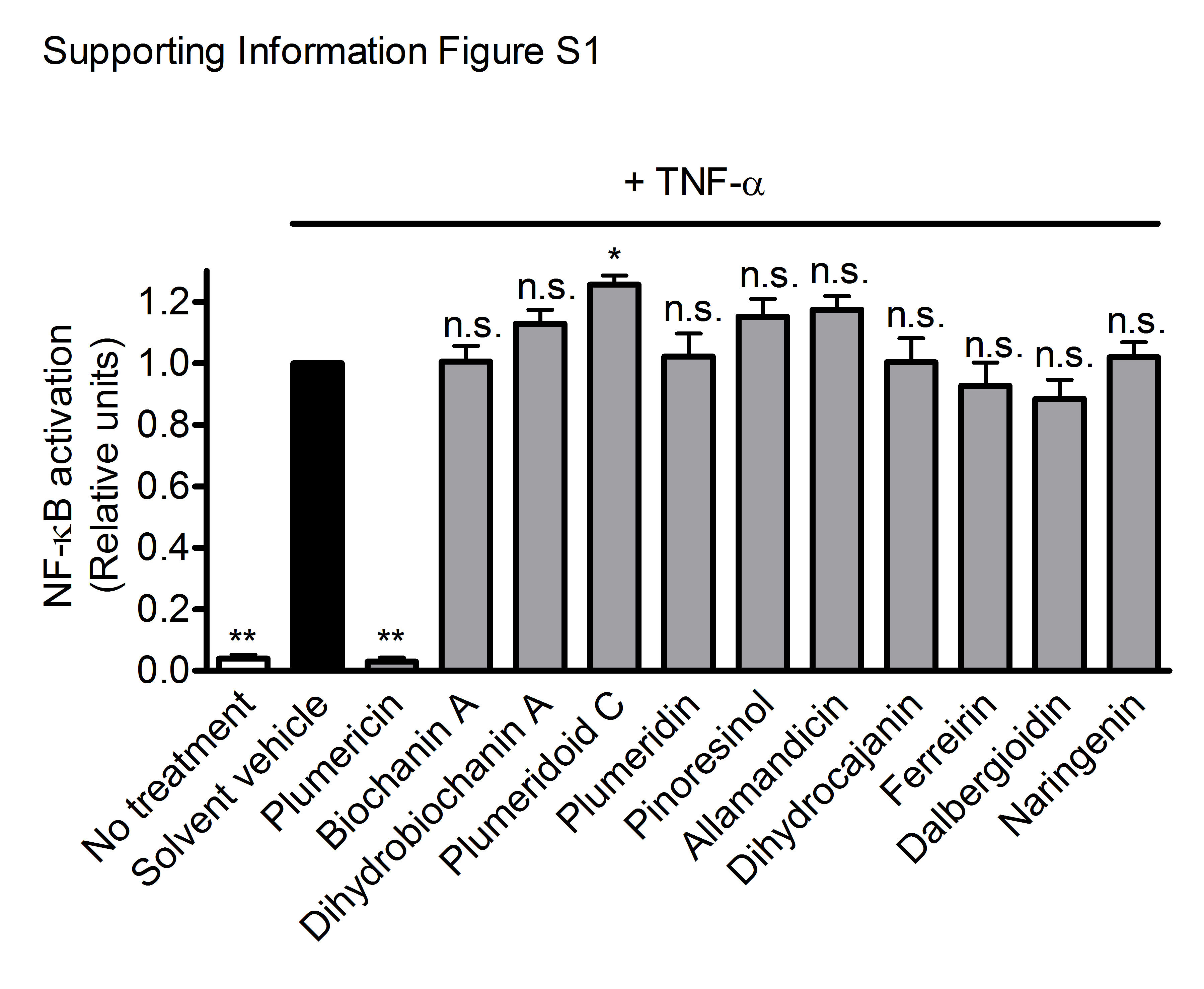

Supplement: Figure S1 — Among all isolated compounds only plumericin is a potent inhibitor of TNF-α-induced NF-κB activation. HEK293/NF-κB-luc cells were pretreated for 30 min with the indicated compounds (10 μMeach) or solvent vehicle (DMSO 0.1%), and stimulated with 2 ng·mL-1 TNF-α for 4 h. The data represent mean ± SEM (n = 3; *P < 0.05, **P < 0.01, n.s. not significant, ANOVA/Dunnett). [file bph0171-1676-sd1.jpg]

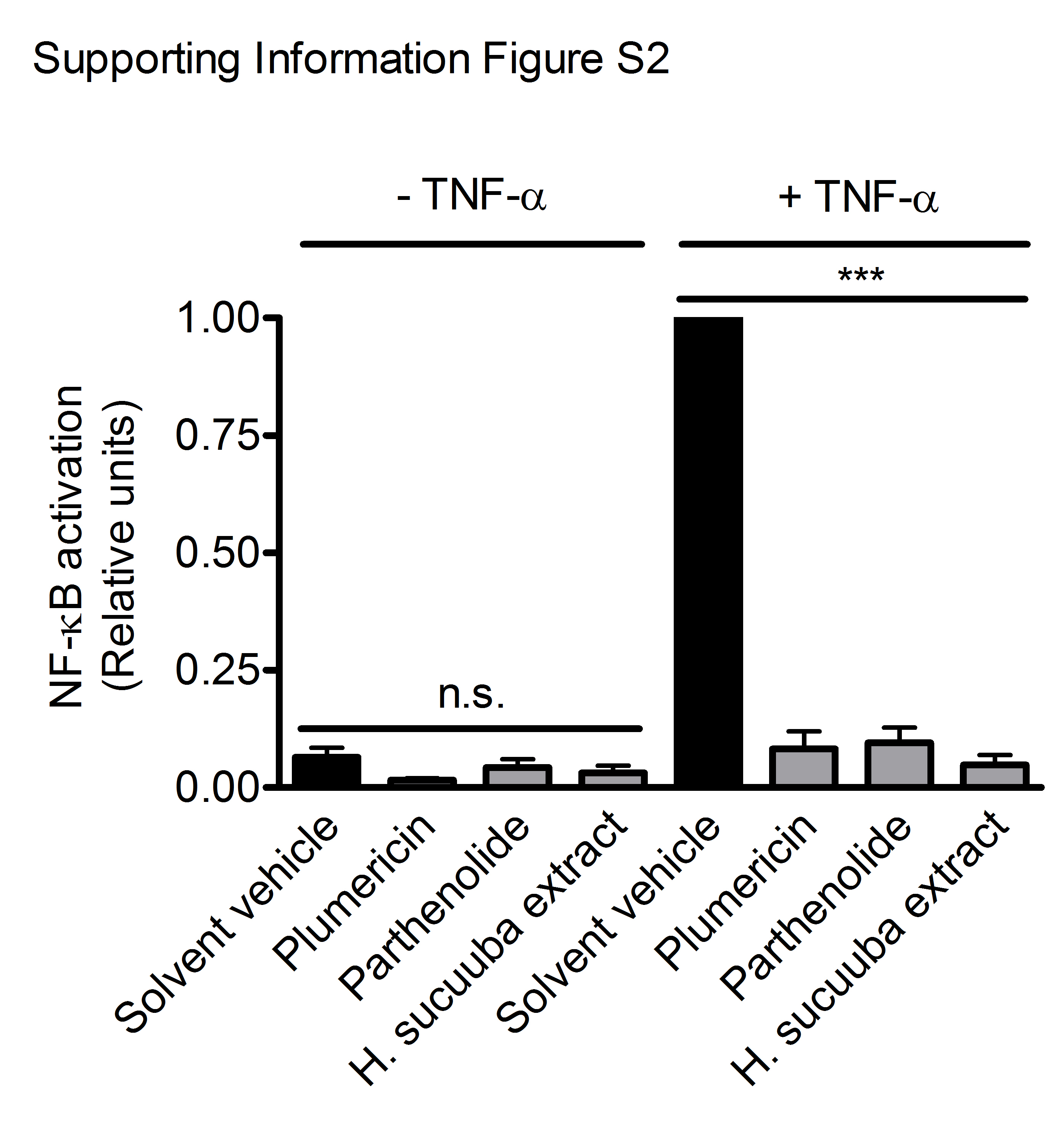

Supplement: Figure S2 — Comparison of the effects of plumericin, parthenolide, and the H. sucuuba extract on NF-κB activity in the presence and absence of TNF-α stimulation. HEK293/NF-κBluc cells were pretreated for 30 min with H. sucuuba extract (60 μg·mL-1), plumericin (5 μμ), parthenolide (5 μμ), or solvent vehicle (DMSO 0.1%), and stimulated with 2 ng·mL-1 TNF-α or treated with the same volume of vehicle (cell culture medium) for 4 h. The data represent mean ± SEM (n = 3; ***P < 0.001, n.s. not significant, ANOVA/Bonferroni). [file bph0171-1676-sd2.jpg]

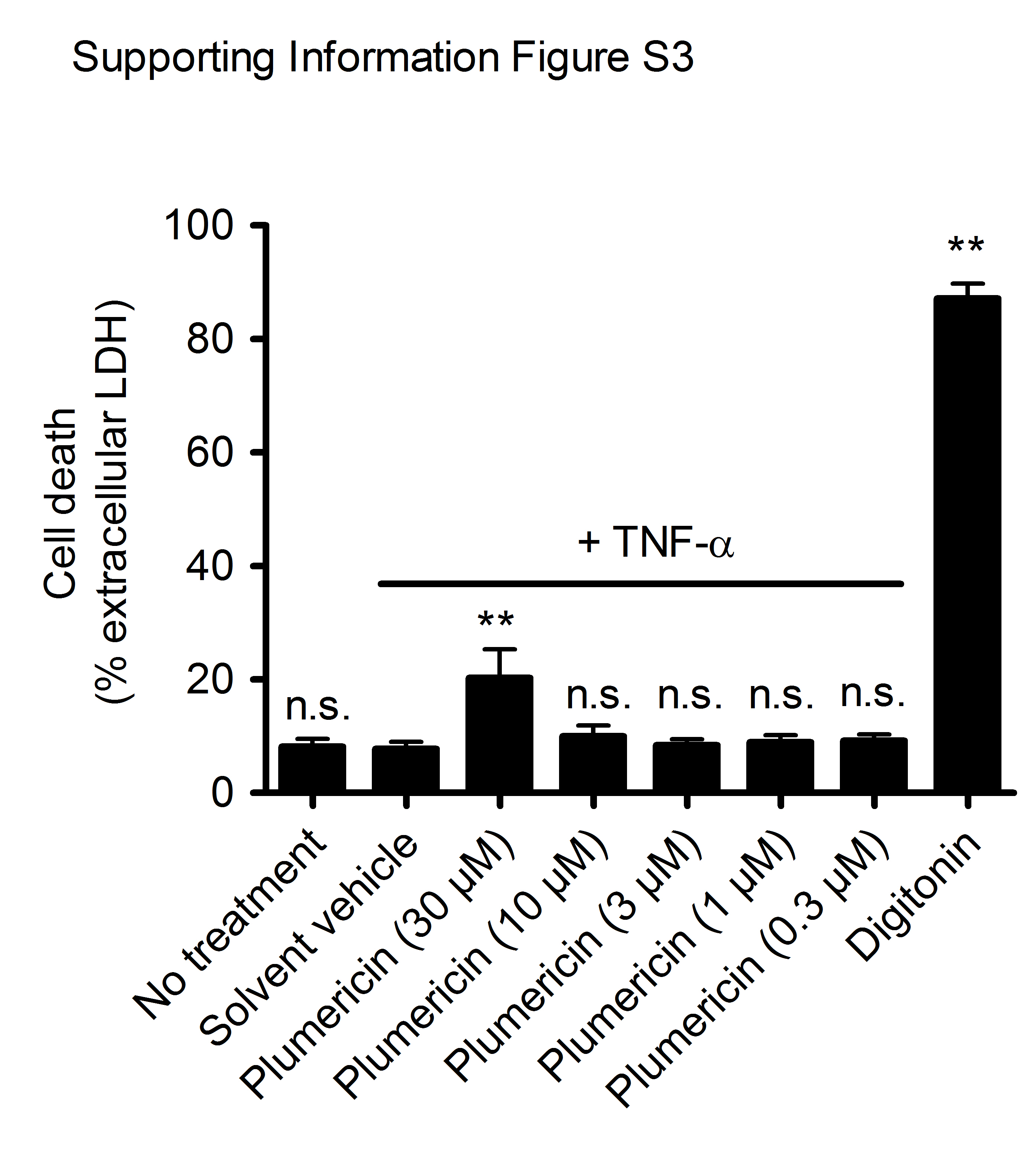

Supplement: Figure S3 — Cytotoxicity determination upon prolonged exposure and in the presence of higher concentrations of plumericin. HEK293/NF-κB-luc cells were treated as indicated in Figure 1A and Figure 1B with solvent vehicle (0.1% DMSO), different concentrations of plumericin, or digitonin (200 μg·mL-1) as positive control. LDH enzyme activity was determined 24 h after the TNF-α stimulation. All data represent mean ± SEM (n = 3; **P < 0.01, n.s. not significant, ANOVA/Dunnett). [file bph0171-1676-sd3.jpg]

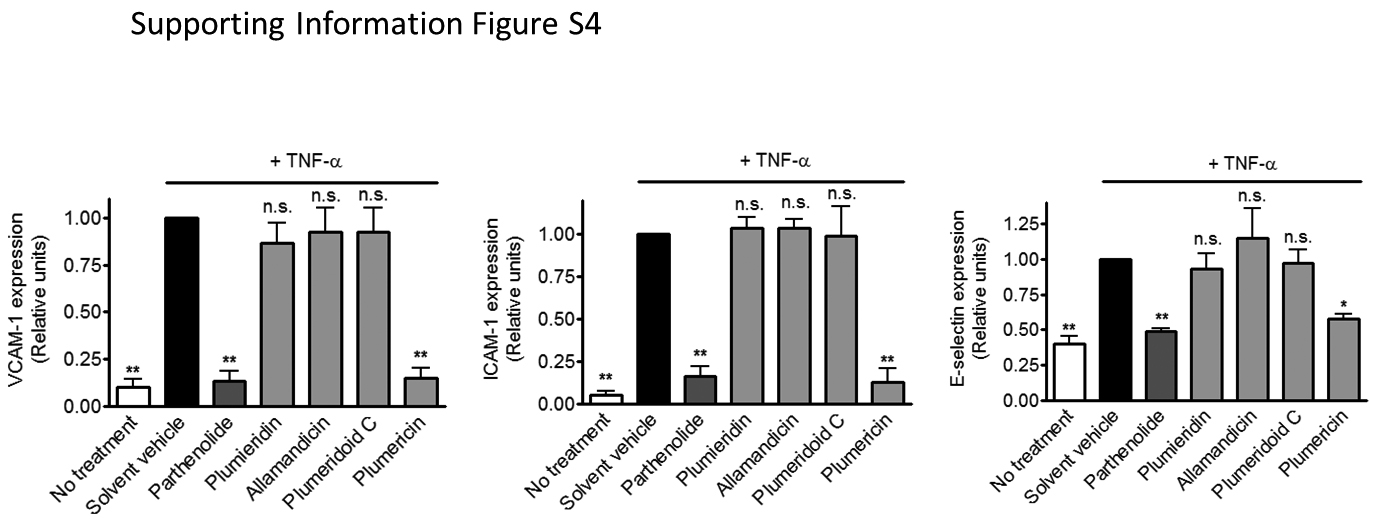

Supplement: Figure S4 — Plumericin but not the other structurally related compounds isolated from H. sucuuba inhibits TNF-α-induced cell surface expression of the endothelial adhesion molecules VCAM-1, ICAM-1, and E-selectin. HUVECtert cells were pretreated with 10 μμ plumericin, parthenolide, plumieridin, allamandicin, plumeridoid C or solvent vehicle (DMSO 0.1%) as control for 30 min prior to stimulation with 10 ng·mL-1 TNF-α for 14 h (VCAM-1, ICAM-1) or 5 h (E-selectin). The protein expression levels were analysed by flow cytometry. Data shown are mean ± SEM (n = 3 for VCAM-1 and ICAM-1, n = 4 for E-selectin; *P < 0.05, **P < 0.01, n.s. not significant, ANOVA/Dunnett). [file bph0171-1676-sd4.jpg]

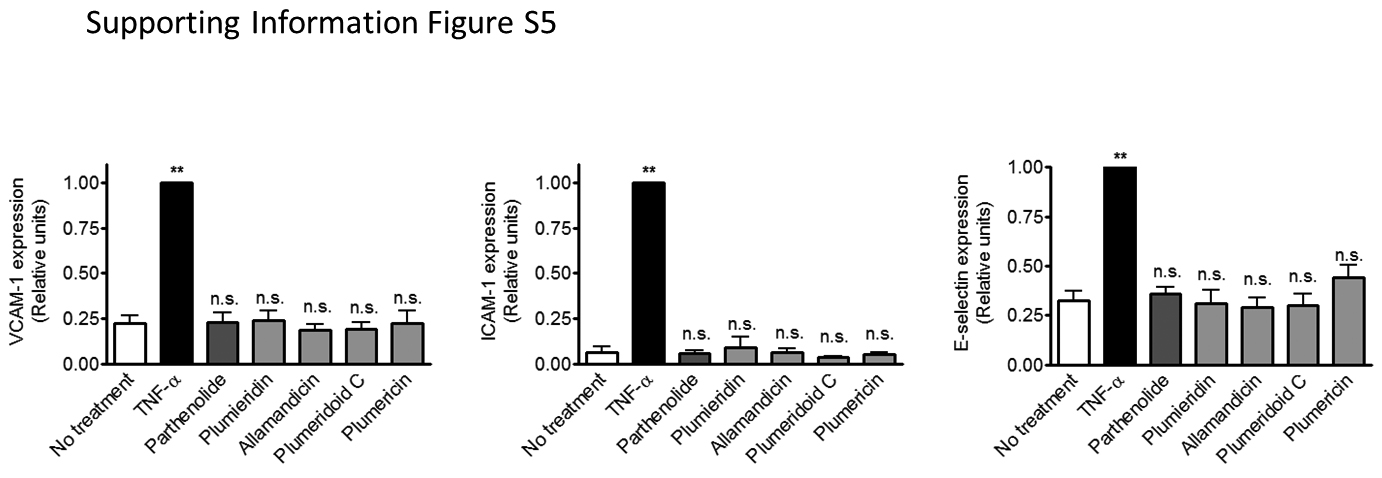

Supplement: Figure S5 — Plumericin and the other investigated compounds do not affect the basal expression of the endothelial adhesion molecules VCAM-1, ICAM-1, and E-selectin in the absence of TNF-α stimulation. HUVECtert cells were treated with 10 μμ plumericin, parthenolide, plumieridin, allamandicin, plumeridoid C or 10 ng·mL-1 TNF-α for 14 h (VCAM-1, ICAM-1) or 5 h (E-selectin). The protein expression levels were analysed by flow cytometry. Data shown are mean ± SEM (n = 3; **P < 0.01, n.s. not significant, ANOVA/Dunnett). [file bph0171-1676-sd5.jpg]

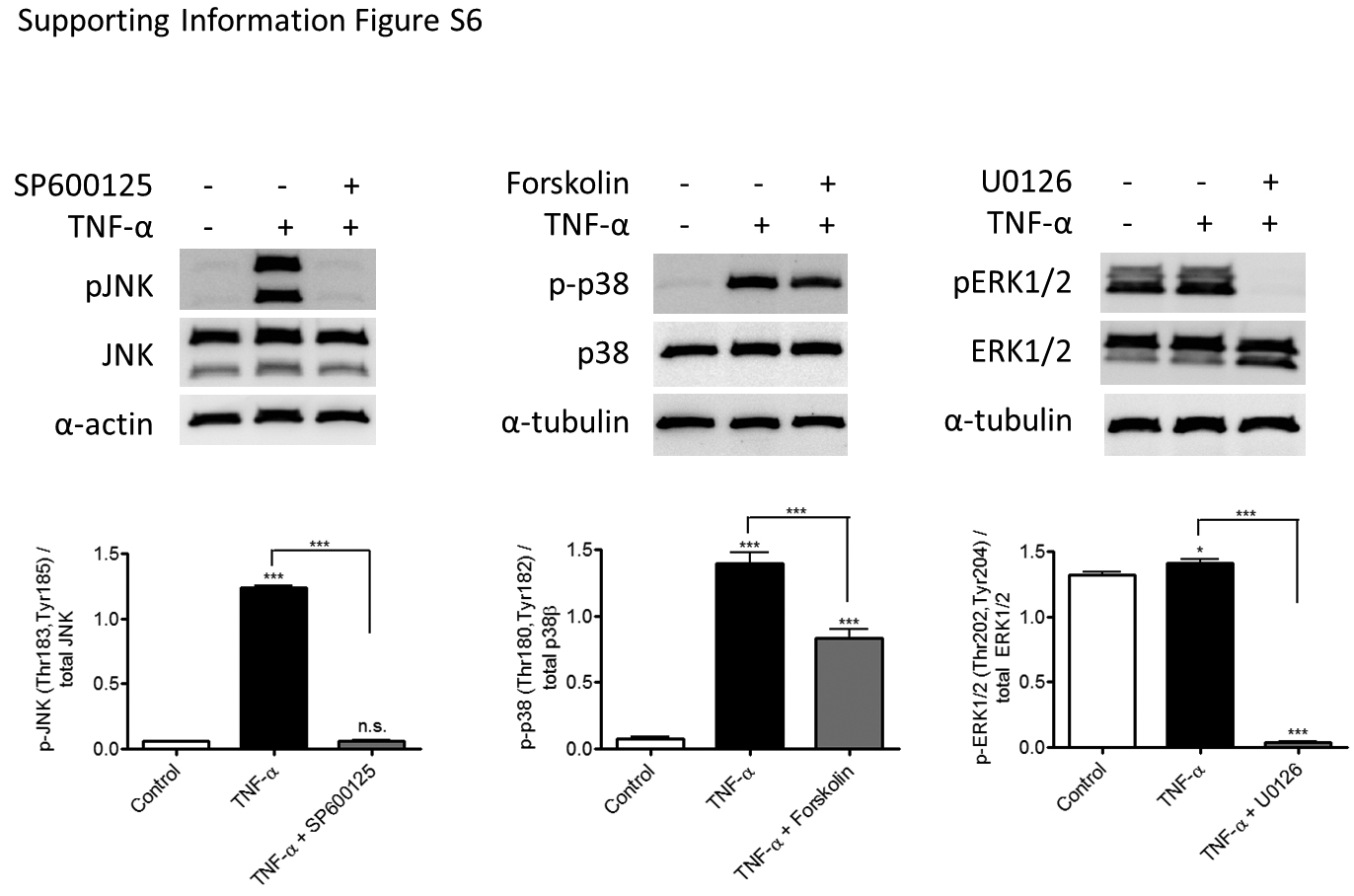

Supplement: Figure S6 — Effect of reference inhibitors on the investigated phosphorylation sites of JNK, p38, and ERK1/2. HUVECtert cells were pre-incubated for 30 min with SP600125 (50 μμ), forskolin (20 μμ), U0126 (10 μμ) or solvent vehicle (DMSO, 0.1% or 0.2%, respectively), prior to stimulation with TNF-α (10 ng·mL-1) for 10 min.Western blot analysis was performed for the total and phosphorylated JNK, p38, and ERK1/2 as described in the Methods section. Tubulin or actin was used as a loading control. Representative blots out of three independent experiments and their quantification as bar graphs (n = 3, mean ± SD, *P < 0.05, ***P < 0.01, n.s. not significant, ANOVA/Bonferroni) are shown. [file bph0171-1676-sd6.jpg]
